# Establishing Wildflower Pollinator Habitats in Agricultural Farmland to Provide Multiple Ecosystem Services

**DOI:** 10.3389/fpls.2016.00363

**Published:** 2016-03-24

**Authors:** C. Sheena Sidhu, Neelendra K. Joshi

**Affiliations:** ^1^Department of Entomology, University of California, RiversideRiverside, CA, USA; ^2^Department of Entomology, University of ArkansasFayetteville, AR, USA

**Keywords:** pollinator, flower plantings, habitats, ecosystem services, agroecosystem, biocontrol

## Introduction

Beneficial insects provide critical ecosystem services and in agriculture their contribution in pollination and pest control is widely evident (Losey and Vaughan, 2006; Kremen and Chaplin-Kramer, 2007). Globally, 35% of food production benefits from pollinator services (Klein et al., 2007). In many systems, pollination has been provided by the domesticated honey bee (primarily *Apis mellifera*), but the reliability of pollination services by wild pollinators is becoming increasingly valued (Garibaldi et al., 2013, 2014). These wild pollinators, the majority of which are bee species, persist independently in the ecosystem by relying on multiple resources to complete their lifecycles (Bohart, 1972). Similarly, natural enemies, such as insect predators and parasitoids, provide vital pest control and also persist independently in the farmscape. Although these beneficial insects are not directly managed for their ecosystem services, the farm landscape surrounding targeted crop fields can be modified to increase their abundance and diversity resulting in increased ecosystem services to support a sustainable agricultural system (Landis et al., 2000; Hannon and Sisk, 2009; Holzschuh et al., 2012).

Managing farmscapes for these wild beneficial insects is especially critical as insects are threatened by human-mediated landscape disturbances (Tscharntke et al., 2005). With wild bee populations in decline (Potts et al., 2010), there is increasing interest in managing for wild bees by incorporating pollinator habitat into farmland. The concept of setting aside land specifically for wildlife within a farmscape is not new (Baudry et al., 2000), however, the addition of wildflower plantings or saving natural wildflower areas is a specific strategy that can be adopted for its multi-functionality in supporting both pollinators and natural enemies. It is especially valuable in that it can be modified and designed to fit specific cropping systems, landscapes, and support the lifecycles of a community of unmanaged beneficial insects. Here we consider how these variables have been examined in recent pollinator habitat studies, and discuss additional considerations to optimize wildflower plantings to benefit multiple ecosystem services.

## Understanding the pollination needs of crops

Supplementary wildflower plantings (adjacent to target crops) function by attracting pollinators from the surrounding landscape to the farmscape and ideally to “spill over” to the crop to provide pollination services (Blitzer et al., 2012; Williams et al., 2015). The purpose of such landscape enhancements is primarily to provide additional nectar and pollen sources for the bee community. In some specific studies, wildflower plantings have been demonstrated as an effective practice for benefiting pollination by increasing crop production (Feltham et al., 2015).

The composition of the wildflower pollinator habitat in farmscapes should depend on the pollination requirements of the crop. One flower-based strategy that may inherently increase pollinators in the farmscape are mass-flowering crops that attract pollinators to the area and may benefit a growing pollinator population by providing a pulse of resources (Le Féon et al., 2013). However, there is the concern that mass-flowering crops can dilute wild bee populations, or there could be competition between crop flowers and concurrently blooming wildflowers (Holzschuh et al., 2008). Also, after a one-time pulse, resources may not be available to support the bee community during the rest of the season.

In cases of mass-flowering crops, additional floral resources should be available and must compliment the crop to be available before and after the crop bloom to extend the full foraging season of the pollinator community (Menz et al., 2011). Timing of the target crop bloom must also be considered, where early, short blooming tree fruit pollinators may need more floral supplements than the pollinators of summer crops, such as annual vegetables and fruit, when a greater diversity of floral resources is available. Any mismatch in complimentary composition of wildflower availability and crop bloom period may not be effective in benefitting wild pollinator community associated with the crop (Ritz et al., 2013).

## Influence of farm landscape on pollinator habitat

Simultaneously, both the farmscape and larger landscape affect the effectiveness of the wildflower pollinator habitat. Wild bee pollination services may be most effective on small farms (Isaacs and Kirk, 2010), and large farms may not be able to completely rely on wild bees (Klein et al., 2012). Landscape context largely affects the wild bee community that is present, in that a depauperate system may not benefit from additional flowers because few wild bees are present, and a heterogeneous, resource-rich landscape may not benefit from any additional resources because resources are readily available to a diverse bee community, but a simple landscape with fragmented resources and isolated bee communities would benefit most from an enhancement (Tscharntke et al., 2012). To ensure an effective wildflower habitat, a general survey of the area could be conducted to assess the current beneficial insect community, plant diversity, and their relative abundance. Only with an existing beneficial community can the population be supported to increase ecosystem services.

## Resource requirements of wild pollinators

Most pollinator habitats are focused on floral resource availability to various pollinators, however, these habitat areas may also provide nesting habitat for pollinators (Cane, 2001; Rands and Whitney, 2011). Pollinators move among habitats in the landscape for various resources (Mandelik et al., 2012), but as central-placed foragers, they have limited foraging ranges and nesting habitat must be located within range of crops that require pollination (Ricketts et al., 2008). Pollinator habitats should also include a diversity of appropriate nesting areas, especially because nesting requirements vary greatly based on wild bee natural histories (Cane et al., 2007). Species respond to the landscape at different scales (Steffan-Dewenter et al., 2002; Tscharntke et al., 2005), and therefore, the food and nesting resources must be both spatially, and temporally available to support a robust and healthy ecosystem (Vaughan and Skinner, 2008), and bee populations (Williams and Kremen, 2007; Zurbuchen et al., 2010). Further, maintaining diversity of season-long floral resources in these habitats is essential to support the diversity of bees (Williams et al., 2015). Additionally, sustainable agroecosystems are generally supported by a diverse pollinator community, thus species-specific resources must suit the requirements of multiple species found in that ecosystem (Winfree et al., 2011).

## Optimizing multiple ecosystem services from pollinator habitats

Multi-functionality is key in promoting land use as pollinator habitat (Wratten et al., 2012), and it should be considered as an intentional step in establishing pollinator habitats, not just a secondary consideration. Several studies have documented that setting aside habitat is effective for supporting natural enemies for pest control (Landis et al., 2000; MacLeod et al., 2004; Gontijo et al., 2013), and in certain cases it can be effective within a year of implementation (Walton and Isaacs, 2011). Native wildflower species can also support natural enemy communities (Blaauw and Isaacs, 2015). For example, natural enemies such as parasitoid wasps (Patt et al., 1997), and flower flies (Ramsden et al., 2015) would benefit from floral resources that are available in the pollinator habitat, and flower flies may also provide additional pollinator services (Jauker and Wolters, 2008). Season-long flowering plants in pollinator habitats also attract other wildlife, and creating wildlife habitat in farmscapes may increase crop yields as reported in a recent study (Pywell et al., 2015). Optimization of these habitats by including diverse perennial flowering plants that attract other wildlife, particularly, beetles, butterflies, and birds (e.g., pollinator and insectivore) will likely increase the aesthetic value of the farm as well as resulting ecosystem services. Different habitat designs could be examined to further increase the multi-functionality, such as using flowers that are also nitrogen fixers, or modifying habitat to function like hedgerows to prevention of soil erosion and storm water infiltration in farmland (Burel, 1996). Overall design can be optimized to build resilience to disturbances in order to provide steady ecosystem services, which will contribute sustainable agricultural systems (Foley et al., 2005).

Biodiversity conservation of arthropods is another important benefit of pollinator habitats in farmscapes. These habitats may also be appropriate for protecting and conserving endangered arthropod species by providing them an appropriate ecological niche to reproduce and sustain populations. However, such benefits are yet to be documented. Recent field research suggests that the negative effects of pesticides on pollinators can also be mitigated by habitat and landscape that supports wild pollinators community in farmland (Otieno et al., 2015; Park et al., 2015), therefore these pollinator habitats might also serve as potential buffer zones for beneficial species in intensive agriculture. However, such spillover benefits of these plantings would also depend on their location as well as pesticide programs of the farms.

## Barriers in implementation: things to consider before establishing pollinator habitats in farmland

In implementing pollinator habitat in the agroecosystem there is the concern of removing land from production, and growers must consider the cost vs. benefits of such habitat, plus additional establishment, and maintenance costs (Landis et al., 2000; Blaauw and Isaacs, 2015). Including the added benefit of multi-functionality can increase the value of establishing wildflower habitat. There is the concern that setting aside habitat could also support greater pest populations, but the associated increased natural enemy population can be effective in suppressing pests (Lee and Heimpel, 2005). While these plantings may have potential to harbor pest population in farmland depending on crop type as well as regional pest problems, further research in this field is needed to better understand how pollinator enhancement plantings impact herbivore populations including various species of pests. Different pollinator habitat modifications and plant species composition could minimize on-farm pest populations, while benefiting pollinator community and other beneficial fauna. Supporting beneficial insects promotes a sustainable agroecosystem as well as ecological interaction among plant and insect species groups (Saunders et al., 2016).

There is also the consideration of time associated with such investments, as it may take several years before pollinator habitat takes effect (Blaauw and Isaacs, 2014a), but the use of different incentive programs, through government agencies such as the United States Department of Agriculture (USDA)—Natural Resources Conservation Service (NRCS) cost share for pollinator habitat creation and maintenance, or Agri-environmental schemes, can help to offset costs (Vaughan and Skinner, 2008; Joshi et al., 2011). In addition to the aforementioned factors, the successful implementation of these plantings could be significantly influenced by the layout and design of plantings and selection of an appropriate location (with optimum distance from the target crops) as well as long-term maintenance. Size of floral plantings could influence pollinator abundance and diversity (Blaauw and Isaacs, 2014b), and it could be a barrier in adoption when growers have limited land availability in their farmland. After initial establishment (with the help of government subsidies or incentive programs), growers will need additional resources to maintain these wildflower pollinator plantings in their farmland, and in long-term, cost associated with the maintenance of these plantings could be a major hurdle in the successful adoption and establishment. However, increased awareness and promoting multi-functionality may increase acceptance and use by growers who will benefit from the multiple ecosystem services provided by the addition of pollinator habitat.

## Recommendations

Most of the studies conducted on pollinator habitats suggest their importance for conserving pollinators (mainly bees) and their ecosystem service in farmland. Fewer studies have investigated the role of these plantings in supporting on-farm biological control and pest control services or other common benefits such as biodiversity conservation. However, in order to maximize the multiple ecosystem services from these habitats in farmland, it is important to examine ecological interactions among various species group, habitat design, and different trade-offs resulting from adoption of this farm practice in agricultural systems. Moreover, examining such interactions, design, and trade-offs will enhance our knowledge toward establishing robust and self-sustained pollinator habitats for sustainable agriculture. Therefore, a comprehensive life-cycle assessment of pollinator habitats and associated overall benefits could be considered as future research areas in this field.

## Author contributions

All authors listed, have made substantial, direct and intellectual contribution to the work, and approved it for publication.

### Conflict of interest statement

The authors declare that the research was conducted in the absence of any commercial or financial relationships that could be construed as a potential conflict of interest.
